# Nanopore-Based Complete Genome Sequence of a Sri Lankan Cassava Mosaic Virus (*Geminivirus*) Strain from Thailand

**DOI:** 10.1128/MRA.01274-19

**Published:** 2020-02-06

**Authors:** Ana M. Leiva, Wanwisa Siriwan, Diana Lopez-Alvarez, Israel Barrantes, Nuannapa Hemniam, Kingkan Saokham, Wilmer J. Cuellar

**Affiliations:** a Virology Laboratory, Agrobiodiversity Research Area, International Center for Tropical Agriculture, Cali, Colombia; b Department of Plant Pathology, Faculty of Agriculture, Kasetsart University, Bangkok, Thailand; c Institute for Biostatistics and Informatics in Medicine and Ageing Research, Rostock, Germany; d Center for Agricultural Biotechnology, Kasetsart University, Nakhon Pathom, Thailand; DOE Joint Genome Institute

## Abstract

Sri Lankan cassava mosaic virus is an emerging pathogen in Southeast Asia. Here, we report the complete genome of a Thai isolate obtained using Nanopore technology. The isolate was collected in 2019 from the northeastern province of Surin, soon after disease eradication was reported in the country.

## ANNOUNCEMENT

Sri Lankan cassava mosaic virus (SLCMV) is a circular, bipartite single-stranded DNA virus belonging to the family *Geminiviridae* (genus *Begomovirus*). It is one of several geographically distinct virus species causing cassava mosaic disease (CMD), a major disease of cassava (*Manihot esculenta* Crantz), in Africa and Asia (1). SLCMV was characterized for the first time in Sri Lanka in 1998 (2); it also occurs in India, and since 2015, it has emerged in Southeast Asia (3, 4). The occurrence of SLCMV in Thailand, the world’s largest exporter of cassava, has not yet been confirmed, although unofficial reports indicate that eradication activities have been taking place in the northeastern region of the country since 2018. Here, we present the complete genome sequence of a Thai isolate of SLCMV, which was obtained using an Oxford Nanopore Technologies MinION sequencer.

The complete genome of SLCMV was obtained from an infected cassava plant collected in the province of Surin in February 2019 from a field presenting a 0.7% incidence of CMD. A phi29 rolling-circle amplification protocol (New England Biolabs, USA) was carried out with 60 ng of total DNA, which had been extracted using cetyl trimethylammonium bromide (CTAB) (5). After a 16-h reaction, 1 μg of the amplified product was randomly sheared using a g-TUBE device (Covaris, USA), and the library was prepared according to the manufacturer’s instructions (genome DNA by ligation sequencing kit, product number SQK-LSK109 [Oxford Nanopore Technologies]). Sequencing was conducted using a FLO-MIN106D (R9.5) flow cell, and base calling was performed in real time using MinKNOW v2.0.

The assembly was performed in two parallel ways (using default parameters), i.e., *de novo* assembly, using the raw Nanopore reads that passed quality control (fastq pass) with Canu v1.8 (6), and reference assembly, using Minimap2 (7) and Pilon (8) with distinct SLCMV genome sequences (isolates Attur 2 [GenBank accession numbers KP455484 and KP455485], Sreekaryam 1 [MK404225 and MK404226], and SLCMV-Col [AJ314737 and AJ314738]). A consensus was created using the reference assembly results, and the quality was checked using Qualimap v2.2.1 (9). The resulting contigs consisted of 72,800 reads for DNA-A, with an average coverage of 15,000×, and 70,681 reads for DNA-B, with an average coverage of 6,000×. The assembled sequences were validated by Sanger sequencing (Macrogen, South Korea) of overlapping amplicons covering the entire circular genome components and were deposited in GenBank.

**FIG 1:**
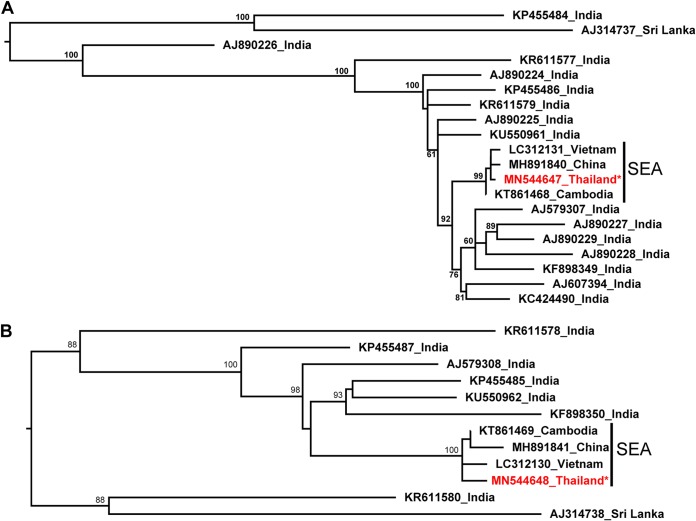
Maximum likelihood tree based on a GTR+G model (confirmed with jModelTest2), using a bootstrap value of 1,000, comparing full SLCMV genome sequences for DNA-A (A) and DNA-B (B) components with those of the Surin1 isolate described in this report (in red). SEA, Southeast Asia.

Sequence Demarcation Tool analysis (10) of the assembled DNA-A (2,759 nucleotides; GC content of 45.9%) and DNA-B (2,737 nucleotides; GC content of 42.7%) showed that both components share ∼99.9% nucleotide identity with other Southeast Asian isolates reported in GenBank. The intergenic regions present a 5′-GGAGA-3′ conserved direct repeat motif known as the iteron, with the corresponding “iteron-related domain” (FRIQSKNIFLTYPKC) at the N terminus of the Rep protein (11). A characteristic of most isolates from Southeast Asia is the presence of a G-to-A transition at position 1569 of DNA-A, which introduces a stop codon that eliminates a 7-amino-acid domain associated with virulence (12) from the C terminus of the Rep protein (AC1). Phylogenetic analysis of all available SLCMV genome sequences using IQ-TREE and jModelTest2 (13, 14) indicated that Southeast Asian isolates form a monophyletic cluster, with >99% bootstrap values, for both DNA components (Fig. 1).

### Data availability.

Genome sequences and Nanopore reads were deposited in GenBank under accession numbers MN544647 (DNA-A) and MN544648 (DNA-B) and in the Sequence Read Archive under accession number PRJNA587722. A phylogenetic tree based on Nextstrain analysis (15) is available online (https://nextstrain.org/community/pestdisplace/CMDASIA?c=virus&r=location).
